# The Role of MRI Pancreatic Protocol in Assessing Response to Neoadjuvant Therapy for Patients With Borderline Resectable Pancreatic Cancer

**DOI:** 10.3389/fonc.2021.796317

**Published:** 2022-01-13

**Authors:** Nervana Hussien, Rasha S. Hussien, Darine Helmy Amin Saad, Mohamed El Kassas, Walid F. Elkhatib, Mai Ezz El Din

**Affiliations:** ^1^ Department of Clinical Oncology, Faculty of Medicine, Helwan University, Cairo, Egypt; ^2^ Department of Radiology, Faculty of Medicine, Ain Shams University, Cairo, Egypt; ^3^ Department of Biological Anthropology, National Research Centre, Cairo, Egypt; ^4^ Department of Endemic Medicine, Faculty of Medicine, Helwan University, Cairo, Egypt; ^5^ Department of Microbiology and Immunology, Faculty of Pharmacy, Ain Shams University, Cairo, Egypt; ^6^ Department of Microbiology & Immunology, Faculty of Pharmacy, Galala University, Suez, Egypt; ^7^ Department of Clinical Oncology, Faculty of Medicine, Ain Shams University, Cairo, Egypt

**Keywords:** borderline resectable pancreatic cancer, ADC, radiological assessment, RECIST criteria, DWI-MRI

## Abstract

**Background:**

Borderline Resectable Pancreatic Cancer (BRPC) remains a unique entity that is difficult to categorize due to variance in definitions and the small number of patients. The ultimate goal is to achieve a free resection (R0) after a favorable response to neoadjuvant therapy that is somewhat difficult to assess by current radiological parameters.

**Aim:**

To evaluate the role of Magnetic Resonance Imaging (MRI) pancreatic protocol, including Diffusion-Weighted Imaging (DWI), in patients with BRPC receiving neoadjuvant therapy, and further compare it to RECIST criteria and outcome.

**Methods:**

Histologically confirmed BRPC patients were prospectively included. DWI-MRI was performed pre- and post-therapy. Clinical characteristics with ensuing operability were recorded and correlated to radiological RECIST/apparent diffusion coefficient (ADC) change, preoperative therapy administrated, surgical resection status, and survival.

**Results:**

Out of 30 BRPC cases, only 11 (36.7%) ultimately underwent pancreaticoduodenectomy. Attaining a stationary or stable disease *via* ADC/RECIST was achieved in the majority of cases (60%/53.3% respectively). Of the 12 patients (40%) who achieved a regression by ADC, 11 underwent surgery with an R0 status. These surgical cases showed variable RECIST responses (PR=5, SD=4, PD=3). Responders by ADC to neoadjuvant therapy were significantly associated to presenting with abdominal pain (p =0.07), a decline in post-therapy CA19-9 (p<0.001), going through surgery (p<0.001), and even achieving better survival (p<0.001 *vs.* 0.66).

**Conclusion:**

DWI-MRI ADC picked up patients most likely to undergo a successful operative procedure better than traditional RECIST criteria. An algorithm incorporating novel radiological advances with CA19-9 deserves further assessment in future studies.

## Introduction

Renowned for its grim outlook, pancreatic malignancies herald a dismal prognosis, with the surgical option serving as the only potential niche for this grave malignancy (1). The emergence of the concept of borderline pancreatic ductal adenocarcinoma (BPDAC) is a small subset of patients that deserves recognition, and many have set out to define this category mainly based on the imaging acquired. Nevertheless, the operating theater acts as the real test if these patients may undergo an actual curative resection or not (2). Because of its excellent accuracy and low complication rate, endoscopic ultrasound-guided fine needle aspiration (EUS-FNA) (or biopsy) is a first-line technique for conclusive tissue diagnosis of pancreatic cancer (3, 4). However, EUS-FNA has some limitations in its diagnostic abilities especially in relatively small tumors, in addition to its limited availability and practice difficulty issues in some resource limited health care settings (5). As radiological diagnostic advances have continued to detect and set the scene for this potentially curative procedure, it remains yet challenging to select those operable cases correctly (6).

A notable quality improvement in detecting and characterization of pancreatic ailments is diffusion-weighted (DW) magnetic resonance (MR). This technique has the added advantage of the relatively quick performance, minus the need for gadolinium-based contrast agents, and offers a measure for tissue diffusion (diffusion coefficients). DW MR imaging utilizes the motion of water molecules in biologic tissues; thus, a restricted signal intensity (or impeded) results in a low apparent diffusion coefficient (ADC) on ADC maps and high signal intensity on DW MR images, and *vice versa* (4).

Therefore, having impeded free water diffusion due to high cell density and fibrosis, a feature of malignancy such as pancreatic carcinoma presents with low ADC compared to healthy pancreatic tissue (7, 8). On the other hand, when water molecules are agile, for example, in necrotic tissue post-treatment, this is reflected by higher ADC values (9). Pancreatic adenocarcinoma has a mean ADC 1.33 × 10^−3^ mm^2^/s with a range of 0.78 ×10^−3^ to 2.32 × 10^−3^ mm^2^/s reflecting the different amounts of cellular density admixed with necrosis and fibrosis (9).

This study aimed to evaluate the role of MRI pancreatic protocol, including Diffusion-Weighted Imaging (DWI) in patients with borderline resectable pancreatic adenocarcinoma after neoadjuvant therapy, to identify responders by MRI with surgical, histopathological, and outcome data.

## Patients and Methods

A prospective study of subjects with BRPC who received their treatment at Helwan and Ain Shams University Hospitals was performed. The study was granted ethical Institutional Review Board approval. The NCCN criteria were used to define Borderline resectable pancreatic cancer as any tumor radiologically in contact with major peripancreatic vasculature as the portal vein (PV) or superior mesenteric vein (SMV) that was deemed resectable (+/− reconstruction) or <180° involvement of the common hepatic artery (CHA) or superior mesenteric artery (SMA) without any tumor extension reaching the celiac axis (CA) or hepatic artery bifurcation (10). Metastatic, resectable, and locally advanced cases were excluded. Treatment naïve patients lacking severe comorbidities with an Eastern Cooperative Oncology Group (ECOG) performance status 0–2 were included. Neoadjuvant chemotherapy (gemcitabine-based or FOLFIRINOX) was administered for six cycles.

EUS-FNA was conducted under deep sedation with intravenous midazolam, propofol, and fentanyl administration, by highly experienced endsonographers in the study centers. Pentax linear echoendoscope EG-3870UTK (PENTAX Medical, Tokyo, Japan, insertion tube of 12.8 mm, biopsy channel of 3.8 mm), with a Hitachi–Aloka Avius processor (Hitachi, Tokyo, Japan), was used for obtaining EUS-FNA. Under EUS guidance, and with the assistance of Color Doppler to exclude interfering vasculature, tissue acquisition was performed using specific EUS needles. The gained material was processed by preserving in 10% neutral-buffered formalin fixative for the creation of a tissue block. The remnant of the aspirated sample was to be smeared on a glass slide and fixed immediately in 95% ethyl alcohol for subsequent staining. All samples were examined by an experienced cytopathologist.

All patients underwent dedicated pancreatic MRI before and after treatment by the fourth week. ADC maps were acquired, and the mean ADC value of the mass was calculated before and after treatment. Also, the longest dimension was measured on T2WI before and after treatment. Vascular relations were assessed on the dynamic study.

### MR Imaging Protocol

The study was performed on a 3.0-T MRI system (MAGNETOM Skyra; Siemens Healthcare, Erlangen, Germany) with an 18-element body phased array coil and a 32-element spine array coil. Before contrast injection, anatomical MRI was performed, including axial T2-weighted (T2W) HASTE (half-Fourier acquisition single-shot turbo spin-echo) with controlled respiration, without and with fat suppression (FS); coronal T2-weighted HASTE without FS; coronal and axial T2/T1TrueFISP; axial 3D T1-weighted Volumetric Interpolated Breath-hold Examination (VIBE) with Dixon reconstruction D (in-phase, out-of-phase, fat-only, and water only images) in breath-holding.

Gadolinium-based contrast was given intravenously using a power injector (Ulrich Medical^®^ Tennessee TM, Germany) at an infusion rate of 1 ml/s. Then, T1-weighted breath-hold VIBE images with SPAIR fat suppression in the arterial, venous, and delayed phases were obtained. Subtracted images were computed as well. Details of sequence parameters are reported in **Table 1**.

**Table 1:** Sequence parameters for MRI pancreatic protocol.

Sequence	TR/TE	Matrix	FOX	Slice thickness	Intersection gap	Acquisition time
**T2-HASTE**	2200/95	320 × 259 mm	350 × 317 mm	5.0 mm	0.1 mm	1.46 s
**T2/T1TrueFISP**	426/1.68	256 × 256 mm	377 × 303.5 mm	5.0 mm	0	0.21 s
**3D T1-VIBE**	4.0/1.31	320 × 182 × 160 mm	400 × 325 mm	3.0 mm	0.6 mm	0.17
**DWI**	7,100/56	128 × 128 mm	380 × 308 mm	4.0 mm	0.08 mm	3.35 s

### Diffusion-Weighted Imaging

DWI was performed using a single-shot echo-planar imaging (EPI) pulse sequence during free breathing. A parallel imaging technique was used to reduce the echo train length. Monopolar gradients were utilized to perform a 3D diagonal encoding with the following b-value(s): 0, 400, and 800 s/mm^2^.

Multidisciplinary consultation was done to assess response and resectability accordingly. Response Evaluation Criteria in Solid Tumors (RECIST) version 1.1 (11) was utilized to measure neoadjuvant therapy effect coupled with MRI ADC value, all through pancreatic protocol MRIs pre- and post-therapy. The pathological completeness of margins (R status) was reported in patients who underwent the procedure.

Data on treatment, response to neoadjuvant chemotherapy by RECIST 1.1, degree of resection (R status), outcome, and survival were collected.

The primary objective was to assess the response rate by RECIST and ADC values utilizing MRI pre- and post-neoadjuvant chemotherapy and then correlate this to the resection margin outcome. The secondary objectives were overall survival (OS) and relapse-free survival (RFS) assessment and their relation to the response parameters (RECIST and ADC value) as well as their relation to the various baseline characteristics.

Overall survival was defined as the time interval between the date of diagnosis and the date of death. The definition of RFS was from the date of diagnosis till the date of cancer recurrence (surgical cases; disease-free survival—DFS)/progression (non-surgical cases; progression-free survival—PFS).

A sample size of 30 patients was selected to achieve an 80% power to detect a mean of paired differences of 0.2 (pre and post mean ADC values) with an estimated standard deviation difference of 0.03 and with a significance value (alpha) of 0.05 based on the work by Dalah et al. (12).

Data analysis and interpretation were conducted using SPSS (Statistical Package for the Social Science; SPSS Inc., Chicago, IL, USA) version 22 for Microsoft Windows. Quantitative data were described as mean ± standard deviation ( ± SD) or median (interquartile range [IQR]) according to data normality, while qualitative data were expressed as frequencies and percentages. According to the data type, the association between data was tested using the Chi-square test with Fisher’s exact, Mann-Whitney test, or one-way ANOVA. Survival data were recorded and tabulated using Kaplan Meier, and the log-rank test evaluated the differences in survival. Variables with a p-value of less than 5% were considered statistically significant.

## Results

A total of 30 patients with histopathologically proven PDAC were recruited, and all received neoadjuvant chemotherapy (gemcitabine-based or FOLFIRINOX). Pre- and post-chemotherapy MRI scans were compared, and after multidisciplinary assessment, 11 patients were deemed operable. Patient baseline characteristics are displayed in **Table 2**.

**Table 2:** General characteristics of the study group (n=30).

Variable		Patients (n = 30)
**Age in years,** mean± SD (median; range)		52.5 ± 6.6 (53.5; 40–62)
**Gender,** No. (%)	Male	22 (73.3)
	Female	8 (26.7)
**Presentation,** No. (%)	Weight loss	26 (86.7)
	Abdominal pain	28 (93.3)
	Jaundice	14 (46.7)
**ECOG performance,** No. (%)	0	11 (36.7%)
	1	19 (63.3%)
**CA19-9 U/ml median (range)**	**Pre-treatment**	250 (100–400)
	**Post-treatment**	170 (20–285)
**Site,** No. (%)	Body	12 (40.0)
	Head	7 (23.3)
	Neck	5 (16.7)
	Tail	6 (20.0)
**Neoadjuvant chemotherapy,** No. (%)	Gemcitabine/cisplatin	11 (36.7)
	FOLFIRINOX	19 (63.3)
**MRI involvement,** No. (%)	Celiac, SMA	1 (3.3)
	Celiac, SMV	3 (10)
	Portal/SMA	10 (33.3)
	Portal/SMV	1 (3.3)
	SMA	5 (16.7)
	SMA, celiac, SMV/PV	1 (3.3)
	SMV/PV, SMA	9 (30)
**ADC × 10^−3^ mm^2^/s,** median (range)	**Pre-treatment**	1.3 (1.1–1.4)
	**Post-treatment**	1.4 (1.3–1.7)
**ADC response,** No. (%)	Stationary	18 (60)
	Regressive	12 (40)
**RECIST,** No. (%)	SD	16 (53.3)
	PD	9 (30)
	PR	5 (16.7)
**Surgery,** No. (%)		11 (36.7)
**R0 (n =11),** No. (%)		11 (100)

PV, portal vein; SMA, superior mesenteric artery; SMV, superior mesenteric vein.

**Figure 1:**
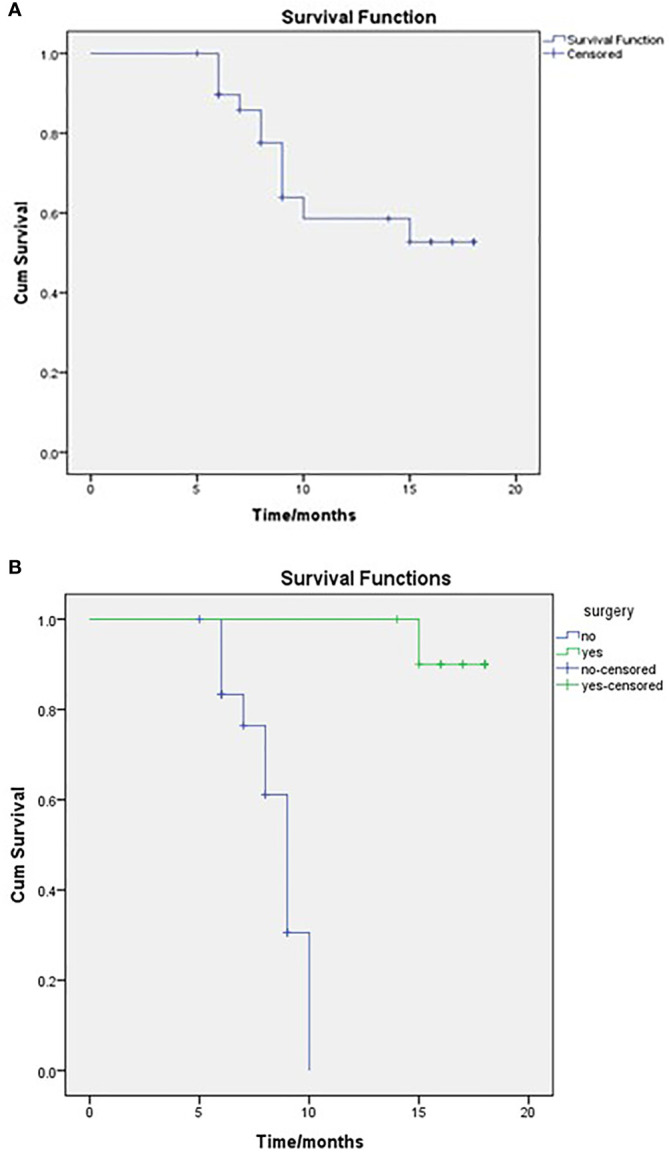
OS of the entire study population **(A)** and comparison between surgical and non-surgical groups **(B)**, as regards to the mean OS for the non-surgical cases was 8.51 months and for the surgical cases 17.7 months. Log-rank, P = <0.001.

After a median follow-up of 14 months (IQR 10.75–22), 19 patients were alive (63.3%), resulting in a mean survival of 13.679 months (SE 1.009; 95% CI 11.702–15.656), while median OS that was not reached (NR) as seen in **Figure 1A**. When comparing survival for the surgical and non-surgical patients, 10 deaths were in the inoperable group, and only one died in the surgical series. The mean OS for the non-surgical cases was 8.51months (SE 0.377; 95% CI 7.77–9.25) and then for the surgical cases, 17.7 months (SE 0.285; 95% CI 17.14–18.25) as also seen in **Figure 1B**. The median OS for the surgical group was NR, and for the non-surgical group, it was 9 months (SE 0.459; 95% CI 8.101–9.899)

RFS in the whole cohort was set at a mean RFS of 10.16 months (SE 1.137; 95% CI 7.934–12.389) and a median of 9 months (SE 1.167; 95% CI 6.713–11.287). The non-surgical series had a mean PFS of 5.88 months (SE 0.576; 95% CI 4.751–7.008) and a median PFS of 5 months (SE 0.483; 95% CI 4.053–5.947).

**Figure 2:**
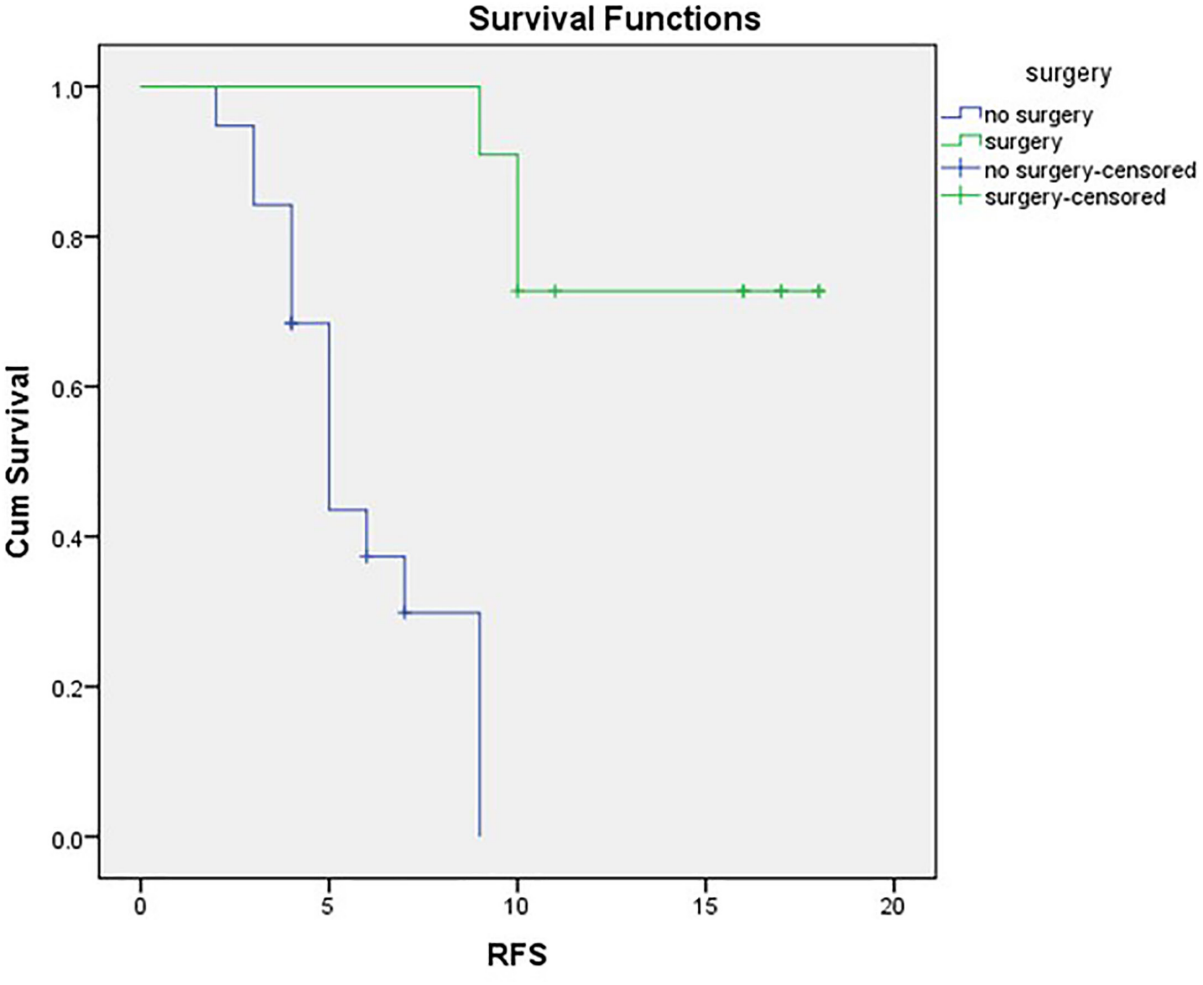
RFS in surgical and non-surgical series.

Mean DFS for the surgical cases was 15.73 months (SE 1.21; 95% CI 13.529–17.925), and with three cases exhibiting recurrence (27.3%), median DFS was not reached, as evident in **Figure 2**.

**Figure 3:**
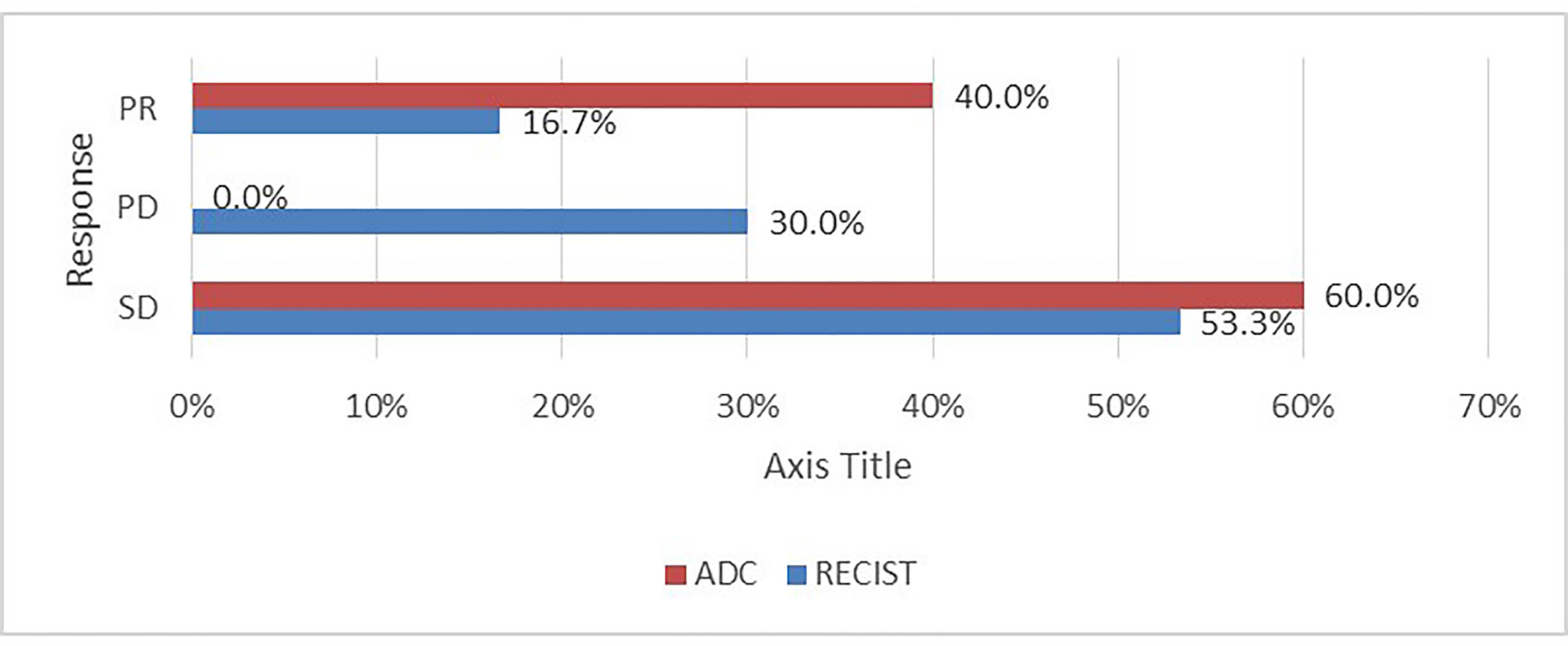
Distribution of response according to ADC and RECIST.

**Figure 4:**
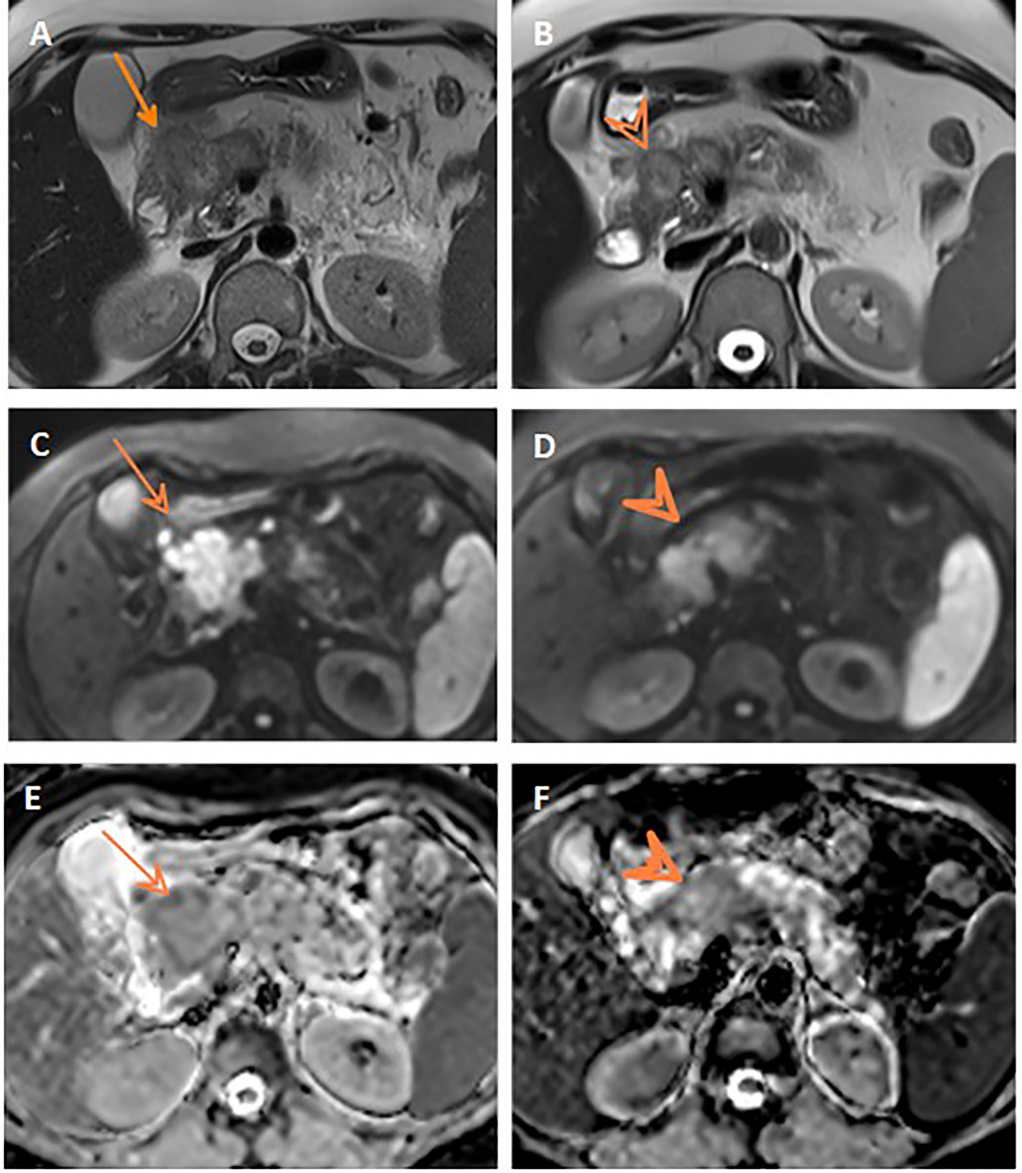
Discordant response between ADC and RECIST. Axial T2WI **(A)** shows progression of the pancreatic mass after neoadjuvant therapy compared to initial axial T2WI **(B)**. Post-treatment and initial DWIs (**C**, **D,** respectively) show corresponding restricted DWI of the mass. Post-treatment and initial ADC maps (**E, F,** respectively) show comparable ADC values of the mass on both studies.

**Figure 5:**
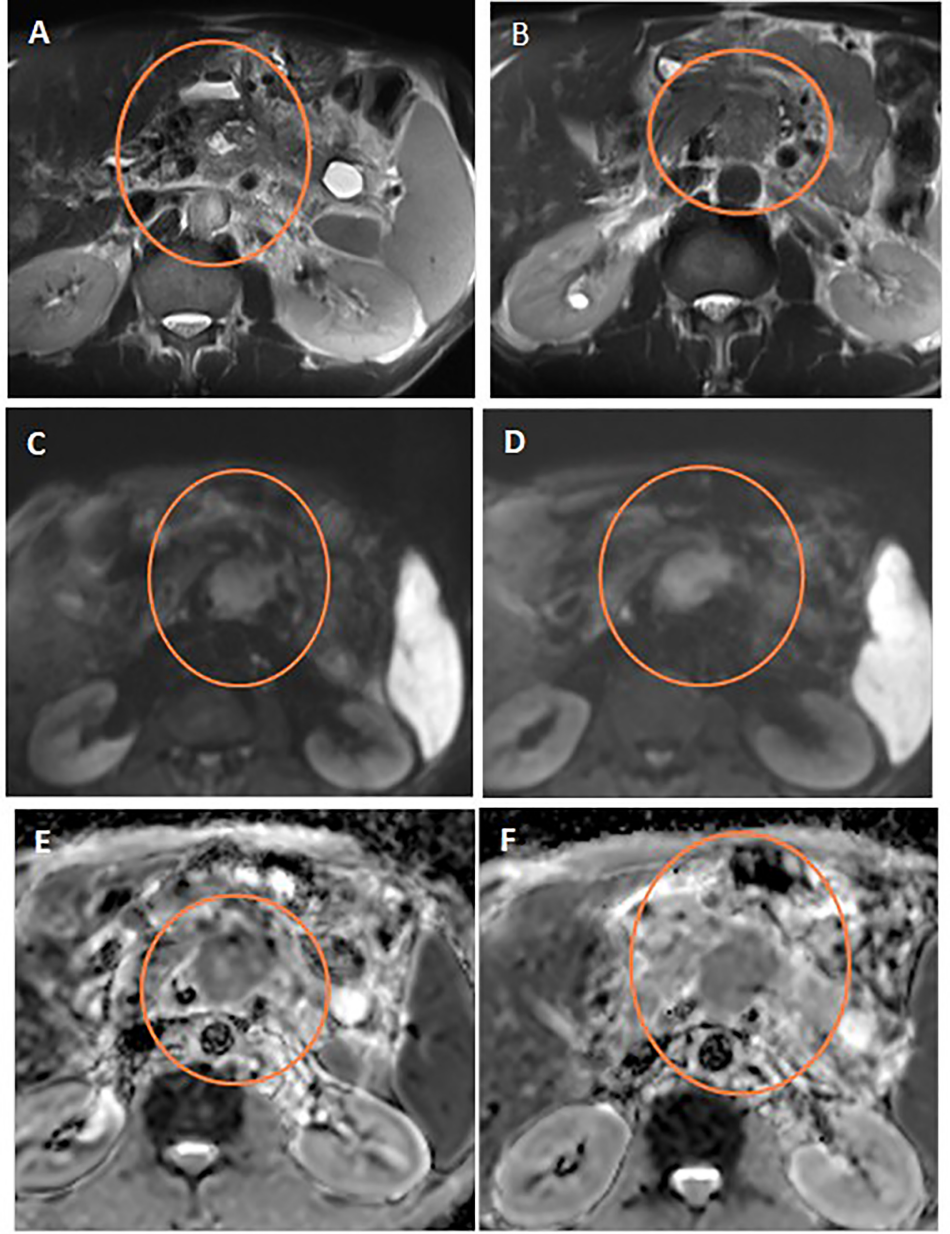
Discordant response between ADC and RECIST. Axial T2WI **(A)** shows stationary size of the pancreatic mass after neoadjuvant therapy compared to initial axial T2WI **(B)**. Note the central cystic change of the mass of necrosis. Post-treatment and initial DWIs (**C, D**, respectively) show corresponding restricted DWI of the mass. Post-treatment and initial ADC maps (**E, F**, respectively) show regression of the ADC values of the mass on post-treatment study compared to initial one.

Assessment for a response *via* RECIST and ADC values is depicted in **Table 3** and **Figure 3**, and it displayed a significant association (p= 0.007). However, it is of poor magnitude based on a kappa statistic of 0.29. The specific ADC value for the resected and non-resected cases is seen in **Table 3**, similarly displaying a significant difference. Discordant response between ADC and RECIST is further depicted in **Figures 4**, **5**.

**Table 3:** Association between ADC response and RECIST.

Variable		RECIST				
		SD (n =16)	PD (n =9)	PR (n =5)	Kappa	P-value
**ADC,** No. (%)	**Stationary (n =18)**	12	6	0	0.293	0.007
	**Regressive (n =12)**	4	3	5		

When examining the median ADC values for all cases pre- and post-NACT, the main driver of a positive correlation overall (P-value 0.001) was more apparent in the surgical cases (P-value 0.003 *vs.* 0.29), as seen in **Table 4**. CA19-9 had a positive statistical significance for all cases, surgical or not, hence did not differentiate the two groups.

**Table 4:** Pre- and post-neoadjuvant ADC and CA19-9 values for the study population.

Variables	Median (range)	Pre-treatment	Post-treatment	P-value
**ADC**	All cases (30)	1.3 (1.0–1.4)	1.4 (1.1–1.7)	0.001
	Surgical cases (11)	1 (1–1.3)	1.4 (1.3–1.7)	0.003
	Non-surgical cases (19)	1.4 (1–1.4)	1.3 (1–1.7)	0.29
**CA19-9 (U/mL)**	All cases (30)	250 (100–400)	170 (20-285)	0.001
	Surgical cases (11)	250 (100–380)	35 (20–48)	0.003
	Non-surgical cases (19)	300 (130–400)	280 (25–380)	0.023

**Figure 6:**
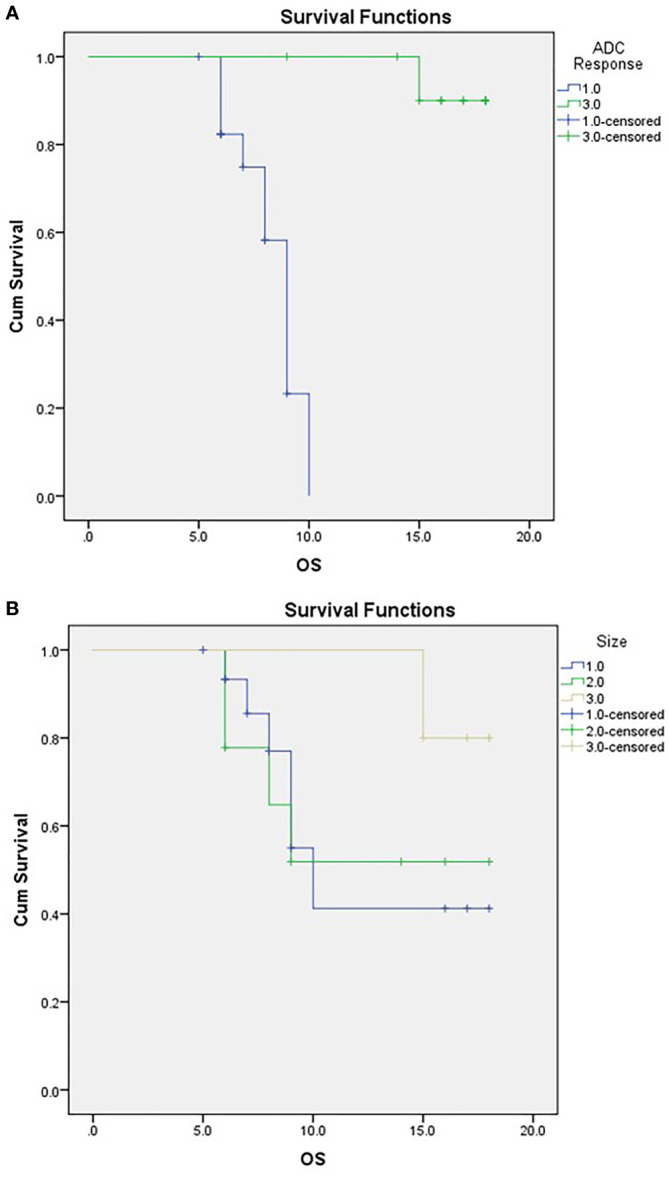
Kaplan-Meier Curve of OS according to ADC response **(A)** and RECIST classification **(B)**. For **(A)** ;1/green=responder, 3/blue=non-responder. For **(B)** 1/blue=PD,2/green:SD, 3/beige: PR.

Attaining a regressive response (or response) to neoadjuvant therapy *via* ADC parameters was significantly associated with abdominal pain as a presenting symptom, a decline in post-therapy CA19-9, and the performance of surgery (**Table 5**). Moreover, ADC displayed significance compared to RECIST criteria when correlated to the outcome, as demonstrated in **Table 5** and **Figure 6**.

**Table 5:** Association between ADC response and characteristics of the study population (n =30).

Variable		ADC Response		
		Stationary (n =18)	Regressive (n =12)	P-value
**Age in years,** mean± SD		53.1 ± 6.9	51.7 ± 6.4	0.58
**Male,** No. (%)		13 (72.2)	9 (75)	0.86
**Presentation,** No. (%)	Weight loss	17 (94.4)	9 (75)	0.13
	Abdominal pain	18 (100)	10 (83.3)	0.07
	Jaundice	8 (44.4)	6 (50)	0.76
**ECOG performance,** No. (%)	0	5(45.5)	6 (54.5)	0.11
	1	7 (36.8)	12 (63.2)	
**CA19-9 in U/ml,** median (Range)	Pre-treatment	280 (130–400)	250 (100–380)	0.36
	Post-treatment	280 (25–380)	35 (20–48)	<0.001
**Site,** No. (%)	Body	7 (38.9)	5 (41.7)	0.98
	Head	4 (22.2)	3 (25)	
	Neck	3 (16.7)	2 (16.7)	
	Tail	4 (22.2)	2 (16.7)	
**Neoadjuvant chemotherapy,** No. (%)	Gemcitabine/cisplatin	8 (44.4)	3 (25)	0.43
	FOLFIRINOX	10 (55.5)	9 (75)	
**MRI involvement,** No. (%)	Celiac, SMA	0	1 (8.3)	0.59
	Celiac, SMV	2 (11.1)	1 (8.3)	
	Portal/SMA	7 (38.9)	3 (33.3)	
	Portal/SMV	1 (5.6)	0	
	SMA	3 (16.7)	2 (16.7)	
	SMA, celiac, SMV/PV	1 (5.5)	0	
	SMV/PV, SMA	4 (22.2)	5 (41.7)	
**Surgery,** No. (%)		0	11 (100)	<0.001

On further analysis for the association between the reduction in CA19-9 after NACT and its correlation with the ADC response, it was apparent that regressive patients had a significantly more significant reduction in CA19-90 (p <0.001) than stationary patients, while RECIST responders lacked this association (p=0.203) (**Table 6**).

**Table 6:** Association between ADC response/RECIST and OS of the study population and CA19-9 (n =30).

Variable		ADC Response			RECIST			
		Stationary (n =18)	Regressive (n =12)	P-value	SD (n =16)	PD (n =9)	PR (n =5)	P-value
**Outcomes,** No. (%)	Alive	8 (44.4)	11 (91.7)	<0.001	10 (62.5)	5 (55.6)	4 (80)	0.66
	Dead	10 (66.6)	1 (8.3)		6 (37.5)	4 (44.4)	1 (20)	
**OS,** mean (95% CI)		8.39 (87.64–9.14)	17.7 (17.14–18.26)	<0.001	12.41 (9.3–15.45)	12.88 (9.19–16.55)	17.4 (16.35–18.45)	0.35
**CA-19-9 in U/ml**	Pre-treatment	280 (130–400)	250 (100–380)	0.36	270 (100–400)	250 (150–370)	250 (100–380)	0.91
	Post-treatment	280 (25–380)	35 (20–48)	<0.001	225 (30–380)	220 (25–380)	33 (20–40)	0.022
	Mean change^a^	14.4 (−1.33–30.8)	210.8 (153.0–267.8)	<0.001	63.4 (3.4–123.5)	78.6 (10.8 146.3)	214.4 (88.9 339.9)	0.203

a Data are presented as mean (95% CI).

Upon addressing the discordance in response, further analysis was performed on the 12 responding patients *via* ADC criteria. They were further categorized into their relevant RECIST subgroup, and CA19-9 normalization values were analyzed. A significant relation was observed for all categories of response by size, thus rendering RECIST criteria inconclusive in response assessment of response even if coupled with the tumor marker (**Table 7**).

**Table 7:** Association between RECIST and CA19-9 in ADC responders.

Variable		ADC Response			P-value
		Regressive (n =12)			
CA19-9		Mean reduction	Pretreatment median (range)	Post-treatment median (range)	
**RECIST**	SD (n =4)	−136.3 (154)	290 (100–380)	35 (30–45)	<0.001
	PD (n =3)	−195.7 (153)	220 (200–250)	45 (35–48)	0.004
	PR (n =5)	−112 (134.1)	250 (100–380)	33 (20–40)	<0.001

## Discussion

The ultimate goal of BRPC is the potentiality of achieving an R0 surgery *via* neoadjuvant therapy. Preoperative therapy has the added advantage of treating micrometastasis at an earlier stage and offers an observation period to exclude rapid progressors exhibiting a poor response to treatment (13).

This study demonstrated the enhanced utility of ADC *via* MRI DWI as a predictor of achieving a favorable pathologic response with clear resection margins paving the way to better survival. This favorable response concurred to achieving normalization in CA19-9 levels as well. Traditional RECIST criteria did not perform well in identifying cases that exhibited response *via* these two metrics.

Further analysis into responding patients by ADC and subclassifying them further by RECIST criteria deemed inconclusive even when CA19-9 response was accounted for with significant p values for all PD, SD, and PR universally expressed.

In the current series, approximately one-third of the BRPC were ultimately resected, 36.6% to be precise, corresponding similarly to the rate reported by two meta-analyses that additionally demonstrated favorable survival rate over 20% at 5-years (14, 15).

The radiologist’s incremental role in selecting neoadjuvant therapy responders has remained difficult to determine despite technological advances firmly. MD Anderson Cancer Center reported that among 122 BRPC patients, the documented CR as assessed by CT was in only one patient (0.8%), PR in 12%, with SD in 69%. Nevertheless, 66% underwent the surgical procedure with a 95% R0 resection plus a 33-month mOS (95% confidence interval, 25.4–40.6 months) compared to a mOS of 12 months (95% confidence interval, 9.5–14.5 months) in those patients that did not undergo the excision. They concluded RECIST criteria 1.1 was not associated with OS and failed to predict resectability (16). Using CT imaging, other studies reported a low response rate that did not signify an abandonment of pancreatectomy (17, 18).

Novel imaging parameters associated with diffusion and perfusion were entered to improve the predictive potential for the operative procedure, pathologic response, and ensuing outcome. A small retrospective study found that tumor delineation by DWI provided the best estimate of tumor size (19). Okada et al. (20) prospectively reviewed 28 patients with BRPC who underwent DWI before neoadjuvant chemotherapy and surgery and found post-treatment whole-tumor ADC value a predictor of R0 resectability; however, the cutoff value of ADC at the location of vascular contact did not discriminate R0 resectability.

Pre- and post-neoadjuvant chemoradiation (nCR) mean ADC values in pancreatic tumors were retrospectively compared and correlated to pathological treatment response in a group of 25 (of which 22 were BRPC) patients by Dalah et al. (12). Significantly higher post-nCR (1.667 ± 0.161×10^−3^) compared with pre-nCR ADC values (1.395 ± 0.136×10^−3^ mm^2^/s) were reported. Additionally, mean ADC after neoadjuvant treatment was significantly associated with the pathological response attained (r=−0.5172; P=0.02) demonstrably higher values in favorably responding tumors. Despite the different methodology demonstrated in their radiotherapy usage and histopathological grading assessment for the response, these results are congruent to ours, whereas we used R0 as a parameter for successful resection.

In another prospective trial, 60 consecutive pancreatic cancer patients were enrolled, and imaging biomarkers as DWI, magnetic resonance spectroscopy (MRS), and PET/MRI correlated stage and PFS (21). This work concluded that these modalities gave complementary data describing the disease characteristics, and a ratio incorporating ADC min served as the most potent biomarker for tumor aggressiveness, stage, and PFS.

Contrastingly, a retrospective observation of 36 pancreatic cancer cases concluded that relying on ADC parameters in response assessment may be misleading and warned against abandoning traditional RECIST criteria. They reported size reduction solely predicted pathologic response with 92% sensitivity and 27% specificity compared to increased ADCs, 48% sensitivity but a better specificity of 73% (22).

Not being devoid of limitations, this study had a small number of patients, and in the end those that were able to undergo the surgical procedure were yet even smaller, as is the case in this borderline subtype. Also, pathologic examination of tissue was not collected, and comment on resection margin sufficed for this parameter, making inter-trial comparisons difficult. However, it is worth noting that R0 alone in our study did provide excellent relevance to improved survival. Finally, ADC has been a subject of interobserver variability according to the region of interest volume and site, not to mention technical factors related to the MRI system (23).

Coming to address this final drawback, radiologists with expertise in abdominal MRI imaging along with rigorous reporting and revision reviewed all scans. Other strengths included the analysis of all recruited cases in intent-to-treat fashion, even though some didn’t undergo the operation. Finally, the majority of cases received FOLFIRINOX, which is considered to have favorable mOS and R0 resection in BRPC, making it a temporally relevant treatment.

## Conclusion

The current study displayed the value of incorporating functional domains to traditional criteria to better elucidate candidates of surgical potential and hence favorable outcome. The simultaneity of response in both assessed imaging reporting modalities in this study was observed in five cases only. Furthermore, in the 12 responders *via* ADC, all attained an R0 operation, and 11 remained alive, indicating that the ADC could be used to assess treatment response for PDAC. Radiomics continues to solve challenging questions in therapy assessment, and relying on old parameters needs to be updated into approved modern evidence-based algorithms and pathways.

## Data Availability Statement

The data that support the findings of this study are available from the corresponding author, upon reasonable request.

## Ethics Statement

The studies involving human participants were reviewed and approved by Helwan IRB. The patients/participants provided their written informed consent to participate in this study.

## Author Contributions

NH was responsible for the conception, design, and quality control of this study. NH, RH, DA, and ME performed the study selection and data extraction, and contributed to the writing of the manuscript. NH and ME collected statistical output and were major contributors in writing the manuscript. MK and WE participated in study selection, editing, and statistical analyses. All authors contributed to the article and approved the submitted version.

## Conflict of Interest

The authors declare that the research was conducted in the absence of any commercial or financial relationships that could be construed as a potential conflict of interest.

## Publisher’s Note

All claims expressed in this article are solely those of the authors and do not necessarily represent those of their affiliated organizations, or those of the publisher, the editors and the reviewers. Any product that may be evaluated in this article, or claim that may be made by its manufacturer, is not guaranteed or endorsed by the publisher.
